# Design of an mHealth App for the Self-management of Adolescent Type 1 Diabetes: A Pilot Study

**DOI:** 10.2196/jmir.2058

**Published:** 2012-05-08

**Authors:** Joseph A Cafazzo, Mark Casselman, Nathaniel Hamming, Debra K Katzman, Mark R Palmert

**Affiliations:** ^1^Centre for Global eHealth InnovationTechna InstituteUniversity Health NetworkToronto, ONCanada; ^2^Institute of Health Policy, Management and EvaluationFaculty of MedicineUniversity of TorontoToronto, ONCanada; ^3^Institute of Biomaterials and Biomedical EngineeringFaculty of MedicineUniversity of TorontoToronto, ONCanada; ^4^Division of Adolescent MedicineThe Hospital for Sick ChildrenToronto, ONCanada; ^5^Department of PediatricsFaculty of MedicineUniversity of TorontoToronto, ONCanada; ^6^Division of EndocrinologyThe Hospital for Sick ChildrenToronto, ONCanada

**Keywords:** Type 1 diabetes mellitus, adolescent, cellular phone, self-care, chronic disease

## Abstract

**Background:**

The use of mHealth apps has shown improved health outcomes in adult populations with type 2 diabetes mellitus. However, this has not been shown in the adolescent type 1 population, despite their predisposition to the use of technology. We hypothesized that a more tailored approach and a strong adherence mechanism is needed for this group.

**Objective:**

To design, develop, and pilot an mHealth intervention for the management of type 1 diabetes in adolescents.

**Methods:**

We interviewed adolescents with type 1 diabetes and their family caregivers. Design principles were derived from a thematic analysis of the interviews. User-centered design was then used to develop the mobile app *bant*. In the 12-week evaluation phase, a pilot group of 20 adolescents aged 12–16 years, with a glycated hemoglobin (HbA_1c_) of between 8% and 10% was sampled. Each participant was supplied with the *bant* app running on an iPhone or iPod Touch and a LifeScan glucometer with a Bluetooth adapter for automated transfers to the app. The outcome measure was the average daily frequency of blood glucose measurement during the pilot compared with the preceding 12 weeks.

**Results:**

Thematic analysis findings were the role of *data collecting rather than decision making*; the need for *fast, discrete transactions*; *overcoming decision inertia*; and the need for *ad hoc information sharing*. Design aspects of the resultant app emerged through the user-centered design process, including simple, automated transfer of glucometer readings; the use of a social community; and the concept of gamification, whereby routine behaviors and actions are rewarded in the form of iTunes music and apps. Blood glucose trend analysis was provided with immediate prompting of the participant to suggest both the cause and remedy of the adverse trend. The pilot evaluation showed that the daily average frequency of blood glucose measurement increased 50% (from 2.4 to 3.6 per day, *P *= .006, n = 12). A total of 161 rewards (average of 8 rewards each) were distributed to participants. Satisfaction was high, with 88% (14/16 participants) stating that they would continue to use the system. Demonstrating improvements in HbA_1c_ will require a properly powered study of sufficient duration.

**Conclusions:**

This mHealth diabetes app with the use of gamification incentives showed an improvement in the frequency of blood glucose monitoring in adolescents with type 1 diabetes. Extending this to improved health outcomes will require the incentives to be tied not only to frequency of blood glucose monitoring but also to patient actions and decision making based on those readings such that glycemic control can be improved.

## Introduction

Chronic disease presents a growing challenge to the health and social care systems in Canada. More than 80% of primary care visits and two-thirds of medical admissions into hospital emergency departments are related to chronic diseases. Effective chronic disease management can result in improved health outcomes and increased quality of life [1].

Wagner [2] and others have proposed a practical framework for chronic disease management. Principally, it proposes that an “informed, activated patient” is needed to provide productive interactions with a “prepared, proactive practice team” [2]. As well, the Health Belief Model introduced the notion of creating a “cue to action” for the patient that elicits health behavior change [3]. Creating such conditions has been used in the design of many chronic disease management interventions across diverse disease populations. One group remains particularly elusive in creating conditions for positive health behavior change, that being adolescents with type 1 diabetes.

Type 1 diabetes mellitus is a chronic condition that is diagnosed in childhood and requires a lifetime of self-management at home and in the community between regular consultations with the health care team. Intensive self-management characterized by frequent self-monitoring of blood glucose is critical in type 1 diabetes to achieve good metabolic control. Improved blood glucose control has been shown to reduce mortality and the incidence of severe and costly complications such as renal and cardiovascular disease. Intensive self-management, including measurement of blood glucose at least 3 times per day, makes it theoretically possible to maintain near-normal blood glucose levels in patients with type 1 diabetes [4].

Despite its importance and a theoretical ability to optimize blood glucose control, worldwide data have repeatedly demonstrated that adolescents do not meet therapeutic targets [5]. Moreover, data from an international study group on childhood diabetes have not demonstrated a correlation between insulin regimen and glycemic control [6], suggesting that factors such as self-care behaviors and educational models likely have substantial impact on outcomes and that increased attention to these factors may lead to improved blood glucose control.

It has been well established that many struggle with interpreting and responding appropriately to the complex data sets that are part of effectively managing type 1 diabetes in real time [7]. Intensive self-management is a challenge for everyone with diabetes, but the mismatch between the demands of intensive self-management tasks and the developmental stage of adolescence often makes self-management even more difficult for this age group and may result in suboptimal glycemic control.

However, there is some recognition and willingness on the part of parents to use technology to assist in the management of their child’s diabetes, particularly when parents have felt that they have some unmet needs with respect to their care [8]. As well, given adolescents’ propensity for new technology, such interventions may provide important opportunities to engage them and to help them improve self-management skills and behaviors [9]. A recent global survey indicated that adolescents around the world are adopting mobile technology faster and in a more immersive way than any previous generation. Recent Pew Internet & American Life Project reports indicate that the mobile phone has become the primary communication tool for the majority of adolescents in the United States; 75% of 12- to 17-year-olds now own mobile phones (up from 45% in 2004) [10]. Text messaging via mobile phone has become the most frequent form of adolescent interaction with friends (overtaking phone calls and face-to-face communication) [10].

The use of the telephone alone, however, has not had much clinical impact. A recent study showed how traditional cognitive behavior interventions such as educational sessions through phone calls had little value in changing negative health behaviors in children with type 1 diabetes [11]. An Australian study of 123 children with an average age of 11.9 years showed that a telephone call intervention by diabetes care providers biweekly did not result in improvements in glycated hemoglobin (HbA1c), diabetes knowledge, psychological parameters, or compliance [12]. A recent systematic review indicated that, although communication technologies may increase the frequency of contact between patient and health care professional, it remains unclear whether this results in improved outcomes [13].

More advanced telehealth methods have not fared better. In a study by Lehmkuhl and colleagues [14], the outcome improvements were unclear and data showed decreased engagement with parents. Youths in treatment reported increased unsupportive and decreased caring parental behavior, although the intervention did improve access to knowledgeable providers and resulted in a clinically significant improvement in glycemic control. There was, however, no significant difference when compared with the control group [14]. Further, a systematic review of type 1 diabetes mobile phone-based interventions indicated that the approach holds great promise, but few studies have shown definitive proof of improved health outcomes in this population [15].

In contrast, in the adult population with type 2 diabetes, a recent randomized controlled trial showed a 1.9% drop in HbA1c in the intervention group using a mobile phone-based remote monitoring and coaching system [16]. The technology used in this and other studies reflects the growing use information and communication technologies (eg, Internet, telephone, mobile phone, and Bluetooth) to track and transmit blood glucose results among adults with diabetes. A systematic review of 17 diabetes telemonitoring studies in adult populations has examined data quality aspects of telemonitoring, effect on clinical outcomes, and impact on behavior of patients and clinicians [17]. Limited work has been done in the development and evaluation of these technologies to enhance self-care in adolescents with type 1 diabetes.

Self-monitoring of blood glucose is critical for effective self-care of type 1 diabetes, but adolescents with diabetes may also require decision-support aids to effectively contextualize a blood glucose result and take appropriate action to optimize glycemic control. The use of remote monitoring technology is likely not enough to elicit positive health behavior in this population. As well, the theoretical foundation of the behavioral intervention that is being delivered should be well established and proven. Heron and Smyth [18] recommended, based on their findings of a systematic review of ecological interventions for health behavior, that mobile technology-based ecological momentary interventions can be effectively implemented for a variety of health behaviors and psychological and physical symptoms. They also recommended the use of ecological momentary interventions that are dynamically and individually tailored and ecologically sensitive [18].

Conclusive methods of engaging the adolescent population in self-care remain experimental and elusive. The present study attempted to engage patients through the use of various approaches including reminders and cueing, social media communication, and the gamification of routine diabetes management tasks. As well, we designed this study to evaluate whether technology can be used to assist adolescents with self-care behaviors with a long-term objective of using technology to improve glycemic control among adolescents with type 1 diabetes.

This study engaged adolescents with type 1 diabetes, their families, and care providers in the design, development, and pilot evaluation of a home- and community-based diabetes telemanagement system. Given the ubiquity of mobile phones as a tool of daily living for many adolescents, we hypothesize that there may be a natural fit between the system and the target population. Hence, the intent of this study was to design, develop, and pilot an mHealth intervention for the management of type 1 diabetes in adolescent children.

## Methods

We employed a user-centered design philosophy to gather requirements and iteratively design the system, leveraging a base remote patient monitoring system that has been tested in other populations. The previous-generation system used BlackBerry smartphones (Research In Motion, Waterloo, ON, Canada) and Bluetooth-enabled medical devices to capture client physiological information and generate and deliver alerts and reports to client, family, and provider [19–21]. Building on this experience, we describe the iterative, user-centered approach to design and develop a new iPhone-based (Apple Inc, Cupertino, CA, USA) system called *bant*.

### User-Centered Design Phase

We employed user-centered design methods that are commonly used in contemporary design and, in particular, consumer-oriented products. This entails the iterative involvement of the end user in the design process by eliciting formal feedback on reference and prototype versions of the intervention; heuristic evaluation by human factors specialists; and formative usability testing of the system.

This design phase involved qualitative, ethnographic interviews with patients and family caregivers (parents) to inform the design and development of the app. In addition, we held focus group sessions with a cross-section of clinical team representatives from the Division of Adolescent Medicine and the Diabetes Program in the Division of Endocrinology at the Hospital for Sick Children (SickKids) in Toronto. Patients aged 12–16 years receiving care at the Diabetes Clinic at SickKids were eligible if they had a diagnosis of type 1 diabetes for more than 1 year; could read, speak, and understand English; and were willing to participate in the study. Each adolescent participant was interviewed individually. Participant parents waited to be interviewed individually after their child. Finally, parent and child were interviewed together to complete the session.

We used an ethnographic research approach when conducting the interviews. Once a sufficient level of saturation was achieved through the focus groups, the recordings were transcribed verbatim. A general inductive method was used in the analysis of the transcripts. Transcripts were read repeatedly and text segments coded for potential themes. As the coding framework developed, transcripts were reanalyzed in light of new themes that may have emerged as a result. Once we completed this step, we derived major themes that were relevant to the research question. Coding was free not to assume any presuppositions. Themes that emerged were used to inform the design and development of the self-management system. A solitary reviewer analyzed the coding, which was validated through member checking of adolescent health and endocrinology specialists at SickKids Hospital.

Data saturation was achieved on completion of the sixth set of patient and parent semistructured interviews. Each interview was conducted by the research coordinator and guided by an interview guide that was based on the study objectives and a priori knowledge of diabetes management, behavior change theory, and health care software design.

### Clinical Pilot

We chose a convenience sample of 20 adolescents to test the assumptions of the intervention. Patients aged 12–16 years receiving care at the Diabetes Clinic at SickKids were eligible if they had a diagnosis of type 1 diabetes for more than 1 year; had received care at the Diabetes Clinic at SickKids for more than 6 months; could read, speak, and understand English; and were willing to participate in the study. Inclusion also required an HbA1c of between 8% and 10% at the time of the previous clinic visit. Information letters describing the study were sent to patients who met the inclusion criteria. The letters identified a mechanism for patients who wanted to opt out so that they would not be approached for recruitment at the time of their next visit. The clinic staff identified the patients to the study project manager at the time of the visit for recruitment. If inclusion criteria were met, and patient and parent agreed to participate, informed consent was obtained. Of the 20 participants, 15 adolescents were given an iPhone 4 to use, and 5 were given an iPod Touch (fourth generation) to use. We felt that this sample of 15 was sufficient to test the assumptions made in this initial iteration of the intervention. The iPod Touch group was added to test whether having a Wi–Fi-only device would affect the user’s experience of the intervention or would alter the patterns of use.

The protocol was approved by the Hospital for Sick Children’s Research Ethics Board (1000017742). As well, we obtained an Investigational Test Authorization (ITA) through Health Canada, due to the advanced technical integration of the blood glucometer with the iPhone app. This was to ensure from a regulatory perspective that the medical device used in the trial was safe. Patients were trained on the system after they consented and were asked to use the system until the time of their next clinic visit, in approximately 12 weeks.

The primary outcome measure was frequency of daily blood glucose readings. The baseline measure was established based on a data extraction of the patient’s personal blood glucose meter at the time of recruitment. We performed a paired *t *test comparing the average number of blood glucose readings 3 months prior to recruitment (baseline) and during the approximately 12 weeks of use of the system during the pilot. HbA_1c_ was also measured at baseline and at the time of the following clinic visit.

We selected well-established measures of diabetes-related self-efficacy, self-care behavior, quality of life, and adherence to self-care psychosocial variables, all of which have well-documented reliability and validity in this population as provided in the references following. Self-care behavior and treatment adherence were assessed at baseline and postintervention using the validated 14-item Self-Care Inventory [22]. Parent–adolescent interaction around diabetes care tasks and decision making were assessed at baseline and postintervention using the Diabetes Family Responsibility Questionnaire [23]. Diabetes-specific quality of life was assessed at baseline and postintervention using the Diabetes Quality of Life for Youth instrument [24].

The study coordinator provided surveys in paper form to participants. Participants completed the survey forms in a secluded area of the diabetes clinic waiting room while they waited for their appointment with their care team. The study coordinator was available in case participants had any questions about the surveys.

## Results

### User-Centered Design Phase

We interviewed 6 adolescents (with 1 parent for each) for this design phase to achieve saturation.

The following major themes emerged and were used to inform the design and development of the self-management system.

#### Theme: Fast, Discrete Transactions

The adolescent participants noted the need for fast transactions taking seconds, not minutes. Several participants commented that social embarrassment was a key factor leading to their avoidance of testing in public (eg, skipping the lunch test in a busy school cafeteria).

#### Design Principle

The design principles for fast, discrete transactions were to (1) ensure that interactions with the system would be fast, (2) use wizards (algorithms used to provide prompts based on available data) to guide user interaction where possible, (3) design the intervention to fit the adolescent lifestyle, and (4) make the interactions related to diabetes discrete.

**Figure 1:**
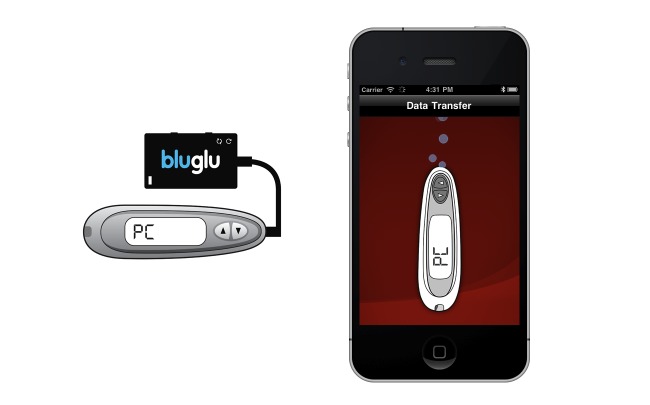
The bluglu adapter for wireless transfer of blood glucose readings via Bluetooth. This avoids the need for manual data entry by the user.

To achieve this goal, we elected to have automated data transfers from their glucometer, rather than having participants enter data manually. We designed an adapter (called bluglu), which allows a OneTouch UltraMini glucometer (LifeScan, Inc., Milpitas, CA, USA) to communicate via Bluetooth, allowing the transfer of blood glucose reading wirelessly, to the iPhone device running *bant* (Figure 1). Data can be transferred from the meter to *bant* at any time. If readings are taken at a time of day (eg, during school lunch) when the iPhone device is not available, data can be transferred to *bant* later in the day at the user’s convenience. However, as soon as the data are transferred, the analysis tools assess the data so that the adolescent gets feedback in real time.

#### Theme: The Role of Data Collecting Rather Than Decision Making

Several participants noted that they did not typically use tracking and analysis tools to review their test results because all of the information was “in the meter.” There seemed to be tension between the roles of tester/collector and analyst/decision maker.

#### Design Principle

Simple data display and decision-support prompts and alerts that integrate into the daily workflow of blood glucose testing may help adolescents to take on more analysis and decision-making tasks in a timely manner, leading to more proactive management.

**Figure 2:**
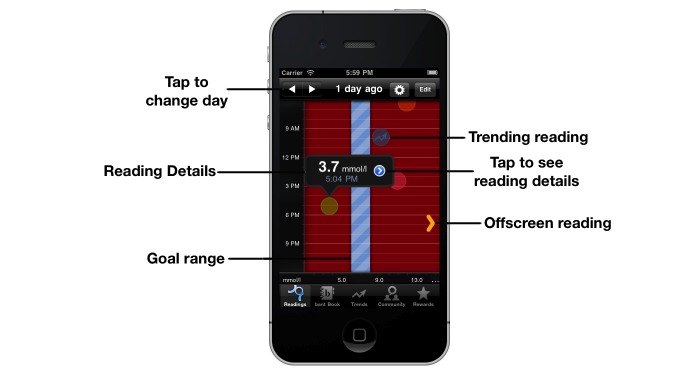
The *bant* Readings screen, indicating the reading through colored circles that represent both value and context.

To address this design principle, we endeavored to give the data greater context and value for decision making. Hence, we designed a novel visual display that has been validated by human factors experts, who specialize in optimizing human–computer interaction, clinicians, app design specialists, and adolescents with diabetes. This provides the user with a summary of daily glycemic control at a glance, associating each data point with context, related to meals and activity, and highlights when blood glucose values are out of range (Figure 2).

#### Theme: Overcoming Decision Inertia

In some cases, adolescents were making few changes to their daily diabetes regimen, even when the blood glucose data had been collected and the profile suggested a change was needed.

#### Design Principle

The design principles to overcome decision inertia were to (1) help adolescents identify blood glucose trends, (2) promote cognitive processing related to identifying and correcting the trend, and (3) integrate rewards and incentives into the system to sustain engagement with the tool.

**Figure 3:**
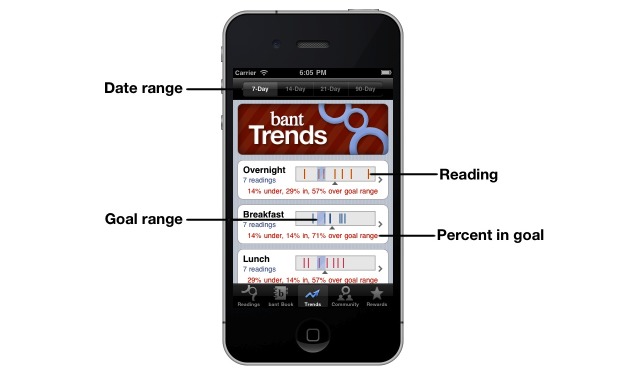
Trends allows at-a-glance review of readings over 7-day, 14-day, 21-day, and 90-day periods. The percentages of readings under, within, and above target range are noted for quick review.

**Figure 4:**
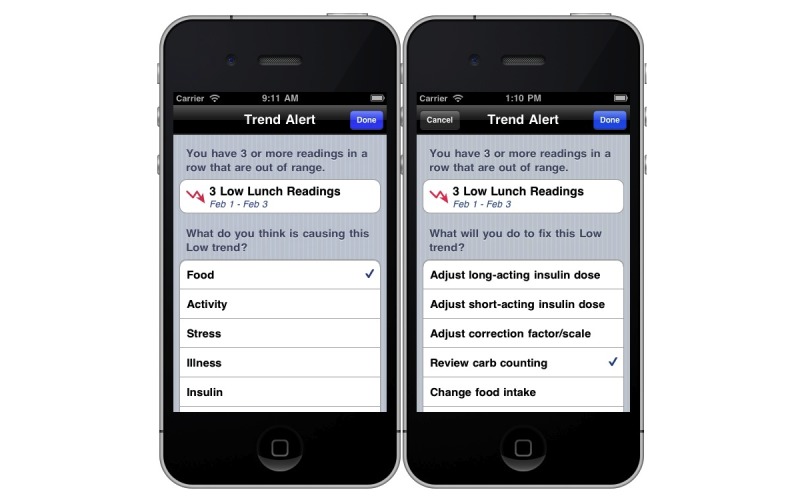
Trend Alert automatically identifies 3-day trends and prompts users to identify what they believe is the cause (left) and their intended action (right).

When blood glucose levels are out of range for 3 days in a row in a particular context (eg, before breakfast), *bant* detects the 3-day trend and prompts the user to make a decision about the cause of the trend and how to rectify it. In addition, *bant* provides data analysis and trending screens that display the percentage of blood glucose levels that are in range at specific times (eg, before meals, before bedtime, or overnight) (Figure 3) as well as decision support via the trend wizard feature, which assists the adolescent in identifying the cause of the trend and the adjustment in regimen that might help improve blood glucose control (Figure 4).

**Figure 5:**
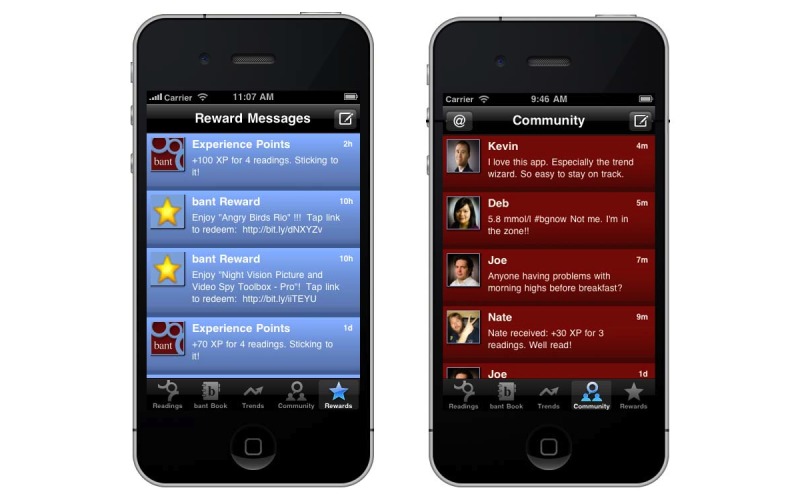
The *bant* Rewards screen (left) indicating experience points (XP) awarded and redeemed for applications and music. The right-hand screen shows the private microblogging community within *bant*, allowing users to communicate with one another.

To further incentivize adolescents to use the app, we designed *bant* with a rewards algorithm that allocated gamelike experience points for adhering to best-practice guidelines for blood glucose testing (goal of three or more tests per day). The algorithm provided increasing point allocations for each test performed (maximum points were awarded for five blood glucose tests performed across five different contexts). Points were awarded based on the instances of each reading and consecutive readings; and bonus points were awarded for a full day of readings (Figure 5). “Leveling-up” was achieved when users had earned 200 experience points, whereby they could be redeemed for Apple iTunes and App Store purchases, usually in Can $1 increments, so the rewards were frequent, up to one reward every 2–3 days for fully adherent users.

As well, we added the ability for users to communicate with their peers in a secure community area of the app to share experiences and gain or provide support (Figure 5). This was achieved through a private microblogging platform similar to the social network Twitter. It used the open-source alternative StatusNet (available at http://status.net/) running on a secure server at our center at Toronto General Hospital. The intent here was for the social aspect of the app to increase regular use of the app and the positive health behaviors it was intended to elicit.

#### Theme: Ad Hoc Information Sharing

Several adolescent participants and parents described an ad hoc approach to day-to-day sharing of diabetes-related information, including test results. Teens generally expressed a willingness to share results with parents and valued their input when it was requested.

#### Design Principle

Ad hoc information sharing would provide opportunities for adolescents to safely share test results and diabetes-related information with parents (as well as peers and clinic staff) via secure online tools and communities for sharing results.

To address this design principle, we elected to integrate the mobile app with a secure online personal health record called TELUS health space, a localized version of Microsoft HealthVault, addressing the needs of Canadian health care practice, including spoken and written language and units. User data could be stored securely and optionally shared with parents and providers. This personal health record is a precertified consumer platform approved by the federal agency Canada Health Infoway. Both patients and providers were provided with health space accounts so as to be able to view shared data from the adolescent.

### Clinical Pilot

The average age of the participants was 14.9 years (SD 1.3 years, N = 20), equally represented by gender (10 each). Of the participants, 9 used an insulin pump and 11 used multiple daily injections.

#### Principal Result: Daily Average Frequency of Blood Glucose Measurement

We recruited 20 patients as planned. Of these, 2 did not complete the full 12-week pilot study and 6 did not have sufficient baseline data from the meters for us to perform the analysis for the primary outcome measure. Hence, we analyzed change in daily frequency of measurements for the remaining 12 patients. For this subgroup analysis (n = 12), the average age of the participants was 15.1 (SD 1.3) years, with 8 girls and 4 boys. Of these participants, 8 used an insulin pump and 4 used multiple daily injections.

Daily average frequency of blood glucose measurement increased 50% (from 2.4 readings per day to 3.6 readings per day, *P *= .006).

#### Principal Result: Rewards

The level of patient engagement in the rewards program indicated the effectiveness of the rewards feature. A total of 161 rewards (an average of 8 rewards each, N = 20) were distributed to patients based on their frequency of measurement, with 50% (10/20) of patients collecting more than 10 awards, 25% (5/20) collecting fewer than 10 rewards, and 25% (5/20) collecting no rewards. There were 2 patients who accumulated a significant number of points and were highly adherent but never redeemed the points for rewards, indicating that the form of reward is not the sole motivator for some patients.

#### Secondary result: HbA1c

HbA1c did not change significantly over the pilot period (average 8.8%, SD 0.74 vs 9.2%, SD 1.03, *P *= .11).

#### Results of Self-Reported Measures

##### Self-care Inventory

We calculated results of the 14-item survey of how well the adolescents followed their prescribed self-care regimen from complete responses from 14 of the 20 participants at baseline and the conclusion of the pilot. The instrument uses a 5-point Likert scale ranging from 1 (never do it) to 5 (always do this as recommended without fail). We found no significant changes in the dimensions of adherence (pretrial average score 3.5, SD 0.93, vs posttrial average 3.6, SD 0.93), blood glucose regulation (average 3.6, SD 0.91 vs 3.6, SD 0.99), insulin and food regulation (average 3.5, SD 0.91 vs 3.6, SD 0.81), and emergency preparedness (average 3.4, SD 1.47 vs 3.4, SD 1.54). There was evidence of improvement in exercise (average 2.9, SD 1.09 vs 3.5, SD 1.15), but it was still not significant (*P *= .069). This finding could be attributed to the seasonal effect of the pilot beginning in the winter and ending in the early spring. The intervention had no specific design aspect for exercise.

##### Diabetes Family Responsibility Questionnaire

Parent–adolescent patient interaction around diabetes care tasks and decision making was assessed at baseline and postintervention. Data for 14 of 20 patients were available. We found slight improvements in both the parent and adolescent scores. None were significant. The average score from the caregivers’ perspective improved from 1.9 (SD 0.14) to 2.0 (SD 0.16). The average score from the adolescents’ perspective improved from 2.1 (SD 0.14) to 2.2 (SD 0.15). A score of 1 indicates the caregiver initiates the responsibility and a score of 3 indicates the child initiates the responsibility. A score of 2 indicates a shared responsibility.

##### Diabetes Quality of Life Instrument for Youth

The 22-item survey results were calculated from complete responses from 14 of 20 patients at baseline and the conclusion of the pilot. We found no significant changes in the dimensions of impact of symptoms (pretrial average 3.4, SD 1.06 vs posttrial average 3.9, SD 1.76), impact of treatment (average 3.7, SD 1.77 vs 3.6, SD 1.33), impact of activities (average 2.9, SD 2.4 vs 3.5, SD 0.24), parent issues (average 8.1, SD 2.71 vs 8.5, SD 2.62), worries about diabetes (average 7.5, SD 4.32 vs 9.0, SD 6.05), and health perception (average 2.2, SD 0.66 vs 2.3, SD 0.68). Generally, most of the dimensions showed either no change or a trend toward worsening in the quality of life.

### Social Networking Findings

A total of 288 posts were made to the community over the pilot period (N = 20). The average was 14 posts per patient over the period. The results were highly skewed with a median of 4.5 and interquartile range of 12.5. As is the case with social networks, a minority of patients posted most frequently: 5 of 20 patients posted 53.1% (153/288) of all posts, and 3 posted only once. Most of the posts were categorized as being either social in nature, accounting for 43.1% (124/288) of the total, or related to the pilot study itself (127/288, 44.1% of the total). The remaining 12.8% (37/288) of posts were categorized as being medically related, including posed questions (12/288, 4%), answers (12/288, 4%), or comments (13/288, 5%).

### Perceptions and Satisfaction

Satisfaction was high, with 88% (14/16 of those who participated in poststudy exit interviews) stating that they would continue to use the system. The remaining 2 patients requested that the app be integrated with their insulin pump (or use a meter that communicates directly with the pump) before continuing to use the system.

## Discussion

These findings revealed specific requirements that were expressed as four themes: the need for *fast, discrete transactions*, the role *of data collection rather than decision making*, overcoming *decision inertia*, and *ad hoc information sharing*. The pilot trial of the resultant intervention showed an improvement in the daily average frequency of blood glucose measurements by 50%. HbA1c did not change significantly. Other self-reported indicators showed no significant results. Satisfaction with the intervention was high, with 88% (14/16 participants) stating that they would continue to use the system.

In the past, several information and communication technologies (eg, Internet, telephone, mobile phone, and Bluetooth-connected blood glucose meters) have been used to track and transmit blood glucose results. However, many of these earlier interventions were developed as electronic logbook applications to collect, store, and display blood glucose readings. Few featured interactive prompts that, as an example, would cue adolescents with diabetes to take action and adjust their treatment protocol. A systematic review of telemedicine interventions to support self-monitoring of blood glucose noted that few studies have examined the relationship between capture of blood glucose data, analysis, advice, and subsequent behavior change. The authors suggest that self-monitoring of blood glucose “with or without telemedicine is only likely to be helpful when test results are linked to educational or behavioural advice and changes in clinical management” [25]. Limited work has been completed on home- and community-based telemonitoring or telemanagement to enhance self-care in adolescents with type 1 diabetes [26]. Furthermore, the authors of this review identified that none of the electronic interventions studied have been particularly effective and that a new approach is needed.

Hence, our approach to the design was not only to capture the end-user requirements of adolescents and parents in managing type 1 diabetes, but also to test new concepts of behavior change. In particular, the use of social networking and rewards were attempts to create adherence to the use of the app, as well as elicit positive health behavior.

The primary outcome of the pilot study was achieved with a significant increase in the daily frequency of blood glucose readings. More frequent self-monitoring of blood glucose (≥3 times daily) is associated with better glycemic control among patients with type 1 diabetes [27]. We hypothesize that the behavioral mechanism that produced this significant result was the combination of the simple automated reminders that were set for the patients at the start of the trial and the issuing of rewards tied directly to taking readings. The use of the rewards system and its apparent effect on eliciting positive health behavior is an example of gamification, which is commonly used in other business sectors, such as commercial patronage loyalty programs. Its application to health care is relatively new. Although games have been used in previous health care behavioral interventions, few examples have demonstrated effectiveness. Rather than creating a diabetes-themed game, we believed that the use of gamelike features of routine self-management tasks would have a greater likelihood of success and could be sustained over longer periods.

For the secondary outcome, however, HbA1c did not change significantly over the pilot period. This result was not entirely unexpected given the small sample size, the short duration of the pilot, and the chronic management problems of the participants involved. Nonetheless, the purpose of the intervention was to elicit positive health behaviors by increasing the frequency of daily blood glucose readings, and we incentivized this behavior, not making changes in the treatment regimen or decreasing the number of out-of-range blood glucose values. In our study, although behavior change related to blood glucose monitoring was achieved, it did not result in improvements in HbA1c; however, previous studies have shown that blood glucose measurement frequency was significantly associated with better metabolic control, with a drop of HbA1c of 0.2% for one additional reading per day (*P *< .001) [28]. We hypothesize that in a properly powered study of sufficient duration that incentivizes not only blood glucose measurements but also metrics of improved glycemic control, HbA1c would improve.

The use of the Trend Alerts aspect of the app is an example of what Heron and Smyth referred to as an ecological momentary intervention [18]. This feature did not show any direct value to health outcomes, however, but we hypothesize that tying reward incentives to actions related to the Trend Alerts may improve health outcomes.

There were no definitive findings from the self-reporting instruments. However, there is some indication that interventions such as *bant* can increase anxiety among patients. This has been observed in other studies by Logan et al in the adult hypertension population [20]. This creates a paradox in that we are attempting to elicit behaviors in patients that will make them more mindful of their condition, yet may have the added negative effect of creating anxiety about their condition. Clearly, the design of the interventions must be cognizant of the potential negative effects, and extra care must be taken in the form of the messaging that is provided to the patient.

We were pleased to learn that schools were very accommodating of the participants when informed of the purpose of the smartphones. In fact, when one was stolen from the locker room, a school principal made an announcement for its return. It was recovered shortly thereafter. No personal information was breached, as a remote wipe command was issued through a device management server when the loss was reported. The device was recovered in its default state.

### Limitations

The pilot was limited to a single-site, small convenience sample with no control group, which limited our ability to generalize the findings and determine the efficacy of the intervention fully. Importantly, the one positive outcome was directly related to the rewards incentive and elicited the expected results. Future iterations of the intervention will attempt to apply this approach to other aspects of patient self-care, leading to a randomized controlled trial.

### Comparison with Prior Work

A recent study of the physically integrated phone and glucose meter GlucoPhone (HealthPia, Paducah, KY, USA) in 40 adolescents with type 1 diabetes showed positive feelings toward the intervention despite having significant technical issues during the 6-month trial [29]. It did not show any improvement in the participants’ quality of life, level of conflict with parents, reported self-management, or glycemic control. This outcome occurred despite significant improvements in health outcomes in the adult type 2 population with the same technology platform previously [30]. Carroll and colleagues speculated that the outcomes with the adolescents were a result of a lack of explicitly attempting to change health behavior. They suggest that adding behavioral contracts could improve the chances of better outcomes [29].

A follow-up study by the same authors showed the additional use of a behavioral contract to be effective in changing the behavior of adolescents with type 1 diabetes [31]. The contract was in the form of a written pledge that the adolescent signed in agreement with the terms set out by the caregiver. The investigators incentivized the adolescent to practice self-care in return for less nagging from the caregiver. The 3-month study was with 10 adolescent–caregiver pairs. Outcomes were improvement in the diabetes self-management profile and significant improvement in HbA1c. However, it is unclear how the behavioral contract is tied to the technological intervention and whether the GlucoPhone was even needed to achieve such a result, other than monitoring adherence to the agreed terms.

Hence, we believe that for the technological intervention to have intrinsic value, the behavioral aspects must be part of the app itself. As nascent as this area of research is, we have found the use of rewards to be effective.

### Conclusions

The use of mHealth behavioral interventions is a new area of research, where there is little data to show whether and how such technology can be used effectively. In our pilot, we demonstrated the feasibility of deploying a diabetes app for use with the adolescent type 1 diabetes population. We also showed that the use of gamification incentives was associated with an improvement in the frequency of blood glucose monitoring in this group. Extending the use of *bant* to improve health outcomes may require that the incentives be tied not only to frequency of blood glucose monitoring but also to patient actions and decision making based on those readings, such that glycemic control can be improved. The long-term sustainability of using iTunes redemption codes as a reward is an issue that requires attention. We would encourage experimentation related to the use of nontangible, virtual rewards as a substitute.

We also have few findings on the effect of the community aspect of the intervention, and its direct value is difficult to ascertain. Only through wider deployment can the use of these social networking aspects be properly evaluated.

Although we cannot fully generalize these results without a control group trial, the findings indicate that the use of these design principles show promise in eliciting positive health behaviors in adolescents with type 1 diabetes.

## Abbreviations

HbA1c: glycated hemoglobin
